# Mitral annular disjunction and high-risk profiles: a conceptual approach to risk stratification and surgical implications

**DOI:** 10.1007/s11748-026-02336-1

**Published:** 2026-06-21

**Authors:** Koji Furukawa, Hirohito Ishii, Shuhei Sakaguchi, Kousuke Mori, Ayaka Iwasaki, Risa Meiri

**Affiliations:** Division of Cardiovascular Surgery, Department of Surgery, Faculty of Medicine, University of Miyazaki, 5200 Kiyotakecho Kihara, Miyazaki-City, Miyazaki 889-1692 Japan

**Keywords:** Mitral annular disjunction, Mitral regurgitation, Arrhythmic mitral valve prolapse, Barlow's disease, Mitral valve surgery

## Abstract

**Objective:**

Mitral annular disjunction (MAD), frequently associated with Barlow’s disease, links degenerative mitral regurgitation to postoperative ventricular arrhythmias and sudden cardiac death, even after technically successful valve repair. However, despite advances in mitral repair, the arrhythmic substrate associated with MAD remains insufficiently addressed in current surgical paradigms.

**Methods:**

A systematic search of PubMed and Google Scholar (2000–2025) was conducted using keywords including “mitral annular disjunction,” “mitral regurgitation,” “arrhythmic mitral valve prolapse,” and “mitral valve surgery.” Of 264 records screened, 41 peer‑reviewed articles, prioritizing high‑impact registries and surgical cohorts, were selected for synthesis.

**Results:**

MAD is associated with paradoxical annular curling and traction‑related myocardial remodeling, which may contribute to fibrosis through pathological stretch. Observational studies suggest that higher‑risk features—such as extensive MAD (≥ 5 mm, particularly ≥ 8.5 mm), late gadolinium enhancement, or syncope—are linked to increased arrhythmic vulnerability. Although modern repair techniques can eliminate anatomical MAD, pre‑existing substrate abnormalities may persist, and ventricular arrhythmias can still occur after technically successful repair.

**Conclusions:**

The extent of disjunction and the presence of associated substrate abnormalities appear to influence long‑term rhythm stability. A conceptual, hypothesis‑generating framework that integrates structural, electrical, imaging, and clinical information may help inform risk stratification. Prospective studies are needed to determine whether a risk‑stratified approach or optimized intervention timing can modify arrhythmic risk or potentially affect long‑term outcomes.

## Introduction

Mitral valve (MV) repair for degenerative mitral regurgitation (MR) is the gold standard [1–3], yet ventricular arrhythmias (VA) and sudden cardiac death (SCD) may still occur postoperatively in a subset of patients [4–7]. This apparent discrepancy between anatomical success and electrical prognosis has drawn attention to mitral annular disjunction (MAD) as a potential mechanistic contributor [5, 7–11].

MAD is defined as a structural separation between the mitral annulus and the left ventricular (LV) myocardium [12–17] and is observed in more than 20% of patients with significant MR [16]. Notably, in Barlow’s disease, MAD is reported far more frequently, with a prevalence of approximately 70–87.4% [16].

However, MAD is insufficiently integrated into current valvular heart disease management [18–20], leaving uncertainty regarding its clinical implications and how this structural abnormality should be addressed in surgical practice. This review synthesizes contemporary evidence to outline the pathophysiology, diagnostic considerations, risk stratification, and management implications of MAD.

## Methods

### Literature search strategy

PubMed and Google Scholar were searched (January 2000–December 2025) using the terms "mitral annular disjunction," "mitral regurgitation," "arrhythmic mitral valve prolapse," and "mitral valve surgery." The search yielded 264 records. After screening titles and abstracts, 41 studies were included. Selection prioritized studies reporting imaging characteristics of MAD, its association with VA or fibrosis, and surgical outcomes. Foundational anatomical descriptions and clinically relevant case reports were also included. Non-English articles and reports without relevance to the structural or clinical aspects of MAD were excluded. No formal quality assessment was performed, consistent with the narrative review methodology.

## Results

### Pathophysiology

MAD represents a structural abnormality that alters the mechanical behavior of the mitral apparatus, with its pathophysiological relevance linked to anatomical separation, abnormal annular dynamics, and traction-related myocardial remodeling [7–11, 21–23]. Understanding these mechanisms is helpful for interpreting the clinical phenotype and considering potential implications for surgical evaluation.

#### Anatomical definition and prevalence

MAD is characterized by a histological separation between the atrial wall–MV junction and the ventricular myocardium, creating a potential space that may contain fibrous or adipose tissue [12–14]. MAD is common in myxomatous MV disease—particularly Barlow’s disease [7, 10, 13, 17, 24, 25].

#### Proposed mechanisms of arrhythmogenicity

**Fig. 1:**
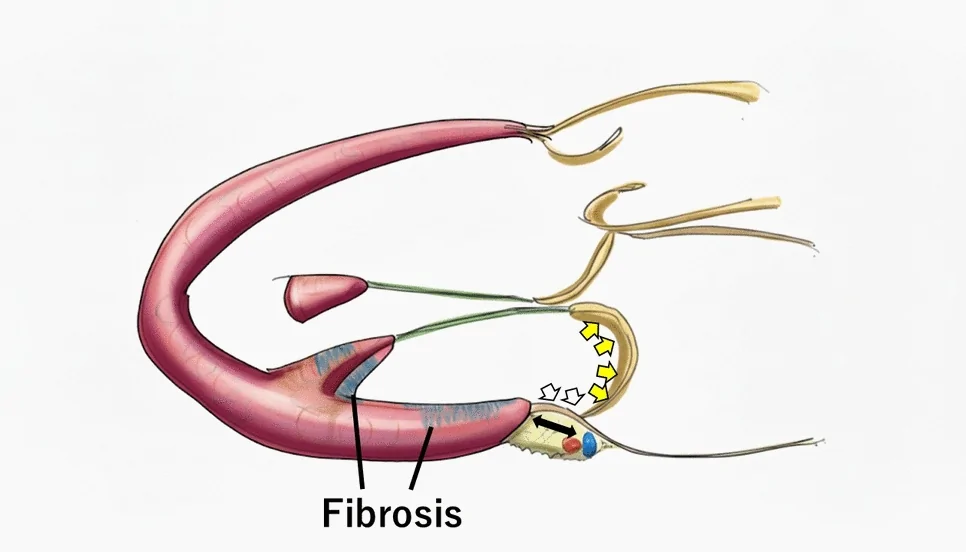
Mechanical cascade of MAD: from paradoxical motion to fibrotic remodeling. Step 1: Paradoxical Curling. Reduced systolic myocardial support allows superior–posterior displacement of the mitral annulus (white arrows). The black bidirectional arrows indicate the annular–ventricular separation characteristic of MAD. Step 2: High-Pressure Loading. Left ventricular pressure (yellow arrow) increases mechanical load on the unstable, prolapsing leaflets. Step 3: Traction and Fibrotic Remodeling. Chronic mechanical load may contribute to traction on the papillary muscles and inferobasal wall, with associated fibrotic remodeling. This substrate may persist even after successful structural repair. MAD, mitral annular disjunction

The arrhythmogenic potential of MAD has been hypothesized to be related to pathological myocardial stretch [8, 9, 21]. Compared with a normally coupled annulus, the decoupled annulus in MAD can exhibit paradoxical systolic curling—moving outward and superiorly [9, 22, 23, 25]—which may impose abnormal traction on the papillary muscles and inferobasal myocardium. Over time, such repetitive mechanical stress could contribute to hypertrophy and replacement fibrosis, potentially creating a substrate for electrical heterogeneity and reentry [8, 9, 11]. Traction-related fibrosis may also extend to the Purkinje network, which could further increase susceptibility to VA [11, 17, 26] (Fig. 1).

#### Clinical relevance

Available data suggest that MAD represents a structural marker of mechanical vulnerability and arrhythmic potential [7–11, 23, 25]. Its identification may therefore inform risk stratification and guide imaging follow-up and rhythm surveillance.

### Diagnosis and risk stratification

Characterization of MAD involves a multimodal strategy integrating structural imaging, electrophysiological markers, and advanced cardiac imaging. These modalities may help describe the extent of annular–ventricular decoupling, electrical instability, and traction-related fibrosis, providing context for identifying patients with higher-risk features.

#### Diagnostic essentials

Transthoracic echocardiography (TTE) remains the primary first-line modality for MAD screening, typically visualized in the parasternal long-axis view at end-systole [10, 17, 21, 25, 27]. Transesophageal echocardiography (TEE) provides higher spatial resolution and can improve delineation of the annular–ventricular interface [10, 22, 23, 28]. Quantitative assessment generally includes measurement of the longitudinal and circumferential extent of disjunction [7, 14, 21, 27, 28].

Cardiac magnetic resonance (CMR) enables myocardial characterization, with late gadolinium enhancement (LGE)—particularly in the papillary muscles or inferobasal wall—reflecting replacement fibrosis [8]. Across observational cohorts, LGE has been one of the most consistently reported markers associated with arrhythmic events [29, 30].

Computed tomography (CT) may also assist in defining annular geometry with high spatial precision [11, 31, 32].

#### Risk stratification framework

Clinical assessment can be organized in a hierarchical manner based on MAD presence, incremental length, and proposed thresholds.

*Presence of MAD*: A disjunction depth ≥ 3 mm is often used as a practical benchmark in TTE [7, 15]. The presence of MAD has been reported as an independent marker associated with adverse outcomes. In a TTE‑based analysis of 595 patients with isolated MV prolapse, MAD was associated with a 2.60‑fold higher risk of clinical arrhythmic events after adjustment for clinical and echocardiographic variables (adjusted HR: 2.60; 95% CI: 1.87–3.62) [29]. In a surgical cohort of 599 patients evaluated with preoperative TTE, preoperative MAD was associated with a 3.33‑fold higher risk of postoperative VA despite technically successful repair (adjusted HR: 3.33; 95% CI: 1.37–8.08) [7]. Additionally, a TTE study of 632 patients found MAD to be associated with increased all‑cause mortality (adjusted HR: 1.81; 95% CI: 1.01–3.25) [33].

*Incremental length*: Observational studies suggest that arrhythmic risk scales with the severity of anatomical separation. In a TTE-based cohort, each 1-mm increase in disjunction was associated with a 16% higher risk of VA (univariable OR: 1.16; 95% CI: 1.02–1.33) [14]. In surgical populations, each 1-mm increase in preoperative MAD length has similarly been associated with a 25% to 35% higher risk of postoperative VA based on multivariable or weighting-adjusted models [7].

*Proposed thresholds*: A 5‑mm threshold has been associated with a 4.23 to 4.53‑fold increase in postoperative VA risk (adjusted HR: 4.23–4.53; 95% CI: 1.70–12.1) in multivariable analyses [7]. A depth greater than 8.5 mm has also been linked to a markedly higher likelihood of nonsustained ventricular tachycardia (NSVT) (univariable OR: 10.0; 95% CI: 1.28–78.1) [14].

Beyond structural measurements, electrophysiological findings provide additional insight into arrhythmic vulnerability. Data from longitudinal surgical cohorts suggest that a 24‑hour Holter‑derived premature ventricular contraction (PVC) burden ≥ 5% or any episode of NSVT may indicate electrical instability [7, 11]. In a large‑scale registry (n = 632), even a 1% PVC threshold was noted to be prognostically relevant, while resting inferior T‑wave inversion (TWI) was associated with a 1.51‑fold increased risk of complex PVC (adjusted HR: 1.51; 95% CI: 1.03–2.22) after adjustment for other risk factors [33]. Seminal studies of sudden cardiac death (SCD) victims (n = 43) reported that 83% exhibited inferior TWI and 100% demonstrated right bundle‑branch block (RBBB)–pattern ventricular arrhythmias [8]. Mechanistically, these ectopic beats—often exhibiting an RBBB pattern with a superior axis—are consistent with an origin from inferobasal structures such as the papillary muscles, suggesting these as potential markers of life‑threatening instability [8, 11].

Tissue characterization with CMR-LGE can identify patchy replacement fibrosis that may contribute to arrhythmic vulnerability [8, 11]. In SCD victims (n = 43), fibrosis was detected in 100% of papillary muscles and 88% of the inferobasal wall, typically with a subendocardial-to-midmural distribution [8]. Clinical data show similar patterns, with LGE present at these locations in 93% of patients with complex arrhythmias compared with 14% without (*p* < 0.001) [8]. Although LGE volume is often small (median 1.2% of left ventricular mass), its localization within the inferobasal wall and papillary muscles may be relevant to arrhythmic risk [8, 11].

Once present, LGE has been reported to offer stronger long-term prognostic information than MAD distance alone [11, 30]. However, the absence of LGE does not necessarily exclude risk [10, 11, 34]. This may reflect the limited sensitivity of current imaging techniques for detecting diffuse interstitial fibrosis or early microscopic scarring, which can still provide an arrhythmogenic substrate in earlier, traction-dominant stages of disease [11].

Unexplained syncope and younger age (< 60 years) may further refine the risk profile [7, 29]. Syncope has been observed more frequently in patients with MAD than in those without (15% vs. 9%, *p* = 0.04), and may serve as a clinical indicator of underlying electrical instability and a higher likelihood of subsequent complex VA [29].

#### Proposed MAD risk profile

Integrating these structural, electrical, imaging, and clinical domains yields a proposed risk profile (Table 1). Recognizing these features and their potential clinical implications may help refine patient assessment and inform surgical judgment when relevant.

**Table 1:** High-risk features of mitral annular disjunction

Feature	High-risk characteristics	Clinical significance (Hazard ratio or %)	References
Morphology	MAD presence	VA risk (adjusted HR:2.60), Post operative VA risk (adjusted HR:3.33), All cause mortality (adjusted HR:1.81)	[7, 29, 33]
	MAD distance ≥ 5 mm	Post operative VA risk (adjusted HR:4.23 ~ 4.53)	[7]
	MAD Distance ≥ 8.5 mm	NSVT risk (univariable OR: 10.0)	[14]
	MAD Distance ≥ 9 mm	Residual MAD risk after mitral annuloplasty (19%)	[10]
Electrophysiology	PVC burden ≥ 5% (24 h) or NSVT	Reported diagnostic criteria for AMVP, used as a key prognostic benchmark in long-term surgical cohorts	[7, 11, 38]
	Inferior T-wave inversion	Risk of complex ventricular ectopy (adjusted HR:1.51)	[33]
	RBBB pattern with a superior axis PVCs	Life-threatening instability	[8, 11]
Tissue Characterization	Presence of LGE at papillary muscles or the inferobasal wall	Identification rate in complex arrhythmias: 93%	[8]
		Marker for long-term arrhythmic risk	[11, 30]
Clinical Profile	Unexplained syncope	Frequently observed in MAD (15% vs 9%), warning sign for underlying electrical instability and a higher risk of subsequent complex VA	[29] [4]
	Younger age (< 60 years)	Greater reduction in post-surgical VA; however, older age (≥ 60 years) shows less benefit	[4]
	Potentially female sex preponderance	Recognized as a clinical indicator frequently observed in malignant AMVP phenotypes	[4]

AMVP, arrhythmic mitral valve prolapse; HR, hazard ratio; LGE, late gadolinium enhancement; MAD, mitral annular disjunction; NSVT, nonsustained ventricular tachycardia; OR, Odds ratio; PVC, premature ventricular contraction; RBBB, right bundle branch block; VA, ventricular arrhythmia

However, current data are derived largely from retrospective studies, and methodological limitations—such as interobserver variability in MAD measurement and discordance across imaging modalities—underscore the need for prospective, multimodality validation of this proposed risk profile.

### Surgical management strategies

Surgical repair of MAD‑associated degenerative MR is generally grounded in standard annuloplasty, which restores annular geometry and provides a stable platform for leaflet coaptation [7, 10, 35].

The choice among rigid, semi‑rigid, and flexible annuloplasty devices remains largely based on surgical preference, although providing adequate mechanical stabilization is an important consideration [8, 17]. To enhance annular support, deep sutures incorporating the ventricular crest have been used to approximate the annulus and reduce the disjunction gap [10, 17]. Although procedural considerations—particularly the proximity of the circumflex artery—must be taken into account, ring fixation reliably reduces the disjunction gap and achieves consistently high anatomical correction rates [7, 10, 35].

### Risk-stratified surgical decision framework

Integrating structural and arrhythmic information may help guide the management of MAD. Structural risk is often associated with a disjunction depth exceeding 8.5–9.0 mm or an extensive circumferential distribution; such features have been reported to predispose to residual MAD when standard annuloplasty alone is applied [10]. In these settings, additional approaches may be considered to support more secure junctional reconstruction [36, 37].

**Fig. 2:**
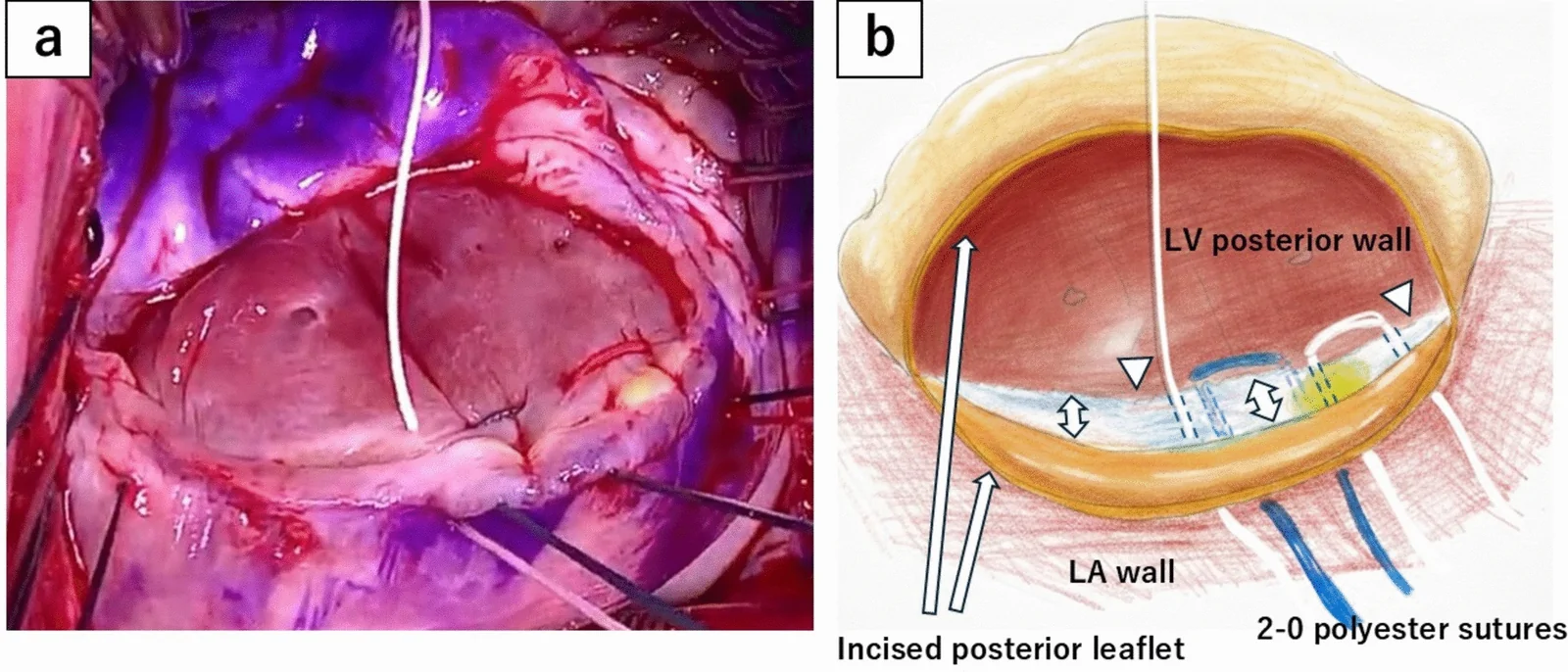
Intraoperative Findings and Schematic Illustration of Targeted Plication for MAD Obliteration. **a** Intraoperative photograph, **b** Schematic illustration. An incision at the base of the posterior leaflet exposes the MAD site (white double-headed arrow) and the left ventricular wall crest (arrowheads). 2–0 polyester sutures are placed through the ventricular crest, exiting the left atrial wall approximately 3 mm on the atrial side of the mitral valve–left atrial junction. Following posterior leaflet repair, the sutures are passed through the prosthetic annuloplasty ring and tied to complete the repair with simultaneous MAD closure. LA, left atrium; LV, left ventricle; MAD, mitral annular disjunction

Figure 2 illustrates the operative view and a schematic representation of complete approximation of the disjunction plane. Conceptually, complete approximation of the disjunction plane may influence annular–ventricular mechanics and reduce local mechanical stress. Nonetheless, these concepts remain speculative, and available evidence is insufficient to determine their impact on MR durability or arrhythmic risk.

The arrhythmic risk axis is characterized by papillary muscle PVCs, LGE on CMR, or a history of unexplained syncope. These markers carry a persistent risk for postoperative VA. For patients with symptomatic or high‑burden VA refractory to medical therapy, catheter ablation is a therapeutic option [11]. Otherwise, concomitant surgical cryoablation of the papillary muscle base and tip under direct vision during MV repair may be considered as a potential adjunct [17]. However, current evidence is limited to early observational experience, and its long‑term impact on arrhythmic outcomes remains uncertain.

**Fig. 3:**
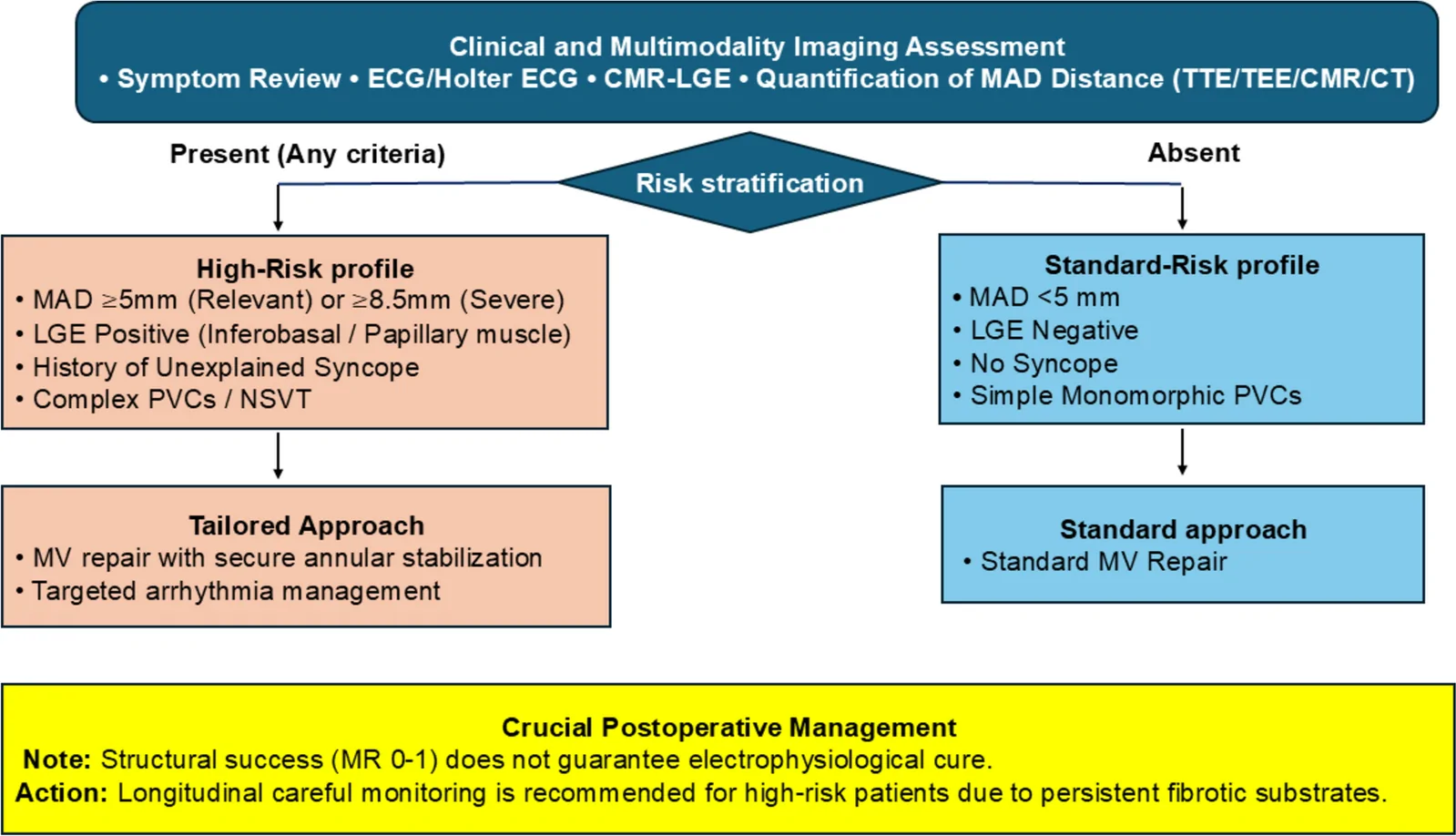
Conceptual, hypothesis-generating framework for MAD-associated risk features and potential surgical considerations. This conceptual model outlines potential distinctions between standard‑ and higher‑risk pathways in MAD. It is hypothesis‑generating and is not intended to serve as a treatment guideline. Any surgical intensification is speculative and exploratory, and the relationship between higher‑risk features and potential procedural choices remains uncertain. Importantly, the arrows represent conceptual relationships rather than directional or sequential clinical pathways. The figure is intended solely to illustrate conceptual domains and does not imply a recommended order of evaluation or intervention. (A) Clinical and Multimodality Imaging Assessment: Initial evaluation includes symptom review (e.g., syncope, palpitations), ECG and Holter ECG for PVC burden and NSVT, CMR‑LGE for myocardial fibrosis, and quantification of MAD distance using TTE, TEE, CMR, or CT. (B) Phenotype Stratification: A higher‑risk profile is suggested when any (OR logic) of the following features are present: relevant MAD (≥ 5 mm), severe MAD (≥ 8.5 mm), LGE positivity, PVC burden ≥ 5%, or unexplained syncope. A standard‑risk profile is considered when all (AND logic) features are absent (e.g., MAD < 5 mm, no LGE, and low PVC burden). (C) Surgical Strategy: In patients with higher‑risk features, secure annular stabilization may be considered. Arrhythmia‑focused adjunctive procedures (e.g., catheter ablation, surgical cryoablation) may be selectively applied in patients with documented arrhythmic vulnerability. (D) Postoperative Management: Ongoing rhythm surveillance is advisable in higher‑risk patients. *Note*: This conceptual model is hypothesis‑generating and exploratory. It uses OR logic for higher‑risk features and AND logic for standard‑risk features. It is not a treatment guideline. CMR, cardiac magnetic resonance; CT, computed tomography; ECG, electrocardiogram; LGE, late gadolinium enhancement; MAD, mitral annular disjunction; MR, mitral regurgitation; MV, mitral valve; NSVT, nonsustained ventricular tachycardia; PVC, premature ventricular contraction; TEE, transesophageal echocardiography; TTE, transthoracic echocardiography

Figure 3 presents a conceptual, hypothesis-generating framework for MAD-associated risk features and potential surgical considerations derived from observational evidence. It is intended to support clinical reasoning and should not be interpreted as a treatment guideline.

### Unresolved issues in MAD management

#### Residual arrhythmic vulnerability after repair

In a surgical cohort of 599 patients evaluated with preoperative TTE, the presence of MAD was associated with a 3.33‑fold higher risk of postoperative VA despite technically successful repair, and each 1‑mm increase in MAD length conferred an additional 25% to 35% increase in postoperative VA risk [7]. In a separate prospective series, 18.6% of patients without baseline arrhythmias developed new‑onset VA after technically successful repair [5]. Together, these observations indicate that postoperative arrhythmic vulnerability can remain substantial, particularly in patients with more pronounced preoperative risk features.

#### Timing considerations in MAD management

Current surgical guidelines primarily base intervention on hemodynamic severity [18–20]. While the 2022 EHRA consensus acknowledges an arrhythmic risk axis for the AMVP/MAD complex [38], MAD and its associated curling dynamic often represent the earliest stage of Barlow's disease, preceding significant MR [24]. In a study by Naksuk et al., patients < 60 years showed a greater VA reduction post‑surgery (OR: 5.8; 95% CI: 1.1–44.7) compared with older cohorts, suggesting that age may modify intervention benefits [4].

These observations have led to the hypothesis that certain high‑risk profiles might warrant earlier consideration for surgical intervention within existing guideline frameworks. However, this remains speculative, and prospective studies are needed to determine whether earlier intervention meaningfully alters arrhythmic or structural outcomes. Balancing traditional hemodynamic triggers with emerging arrhythmic risk stratification therefore represents an area requiring further investigation [11, 34].

#### Minimally invasive surgery in MAD

The advent of robotic platforms has the potential to expand the management of complex MV disease, particularly for the Barlow/MAD phenotype [39, 40]. Studies report a 99% repair rate in robotic Barlow cases, compared with approximately 80% in standard national registries [39]. Magnified 3D visualization and enhanced instrument dexterity may facilitate high‑fidelity suturing within the deep atrioventricular junction, supporting the effective execution of deep suture techniques [39, 40]. Long‑term durability is suggested by reported rates of 92.0% freedom from > moderate MR at 5 years and 99.2% freedom from reoperation at 3 years, indicating that robotic assistance may help standardize MAD‑directed maneuvers within a minimally invasive approach [39, 40].

## Discussion

Emerging data from transcatheter edge‑to‑edge repair (TEER) add further complexity to the interpretation of MAD. Shechter et al. reported regression of MAD in most patients after TEER, with the degree of regression correlating with clinical improvement [41]. This load‑responsive behavior challenges the traditional view of MAD as a fixed structural abnormality. These observations suggest that MAD may behave as a load‑dependent rather than a static lesion. Prospective studies with standardized, multimodality imaging are needed to clarify these mechanisms, validate proposed thresholds, and better define the prognostic and therapeutic implications of MAD.

### Limitations

Current evidence on MAD-associated arrhythmic risk is limited by the retrospective design of most available studies and by inconsistent definitions of MAD across imaging modalities. The risk associations summarized in this review are therefore predominantly observational, and no randomized data exist to establish causality or to determine whether modifying anatomical or mechanical features alters arrhythmic outcomes. Surgical series may also be affected by selection bias, as referred patients often have more advanced disease, and reports of enhanced repair techniques or papillary muscle cryoablation are based on small sample sizes without prospective validation. As a result, the clinical relevance of these procedural strategies remains uncertain.

## Conclusion

Standard mitral repair restores valvular competence, yet MAD may continue to contribute to arrhythmic vulnerability in selected patients. Effective management may therefore benefit from integrating anatomical, mechanical, and electrophysiological perspectives. The extent of disjunction and the presence of associated substrate abnormalities appear to influence long‑term rhythm stability.

Within this framework, identifying patients with higher‑risk profiles may help inform surgical decision‑making, although its clinical utility remains unproven. Prospective studies are needed to determine whether a risk-stratified approach or optimized intervention timing can modify arrhythmic risk or potentially affect long‑term outcomes.

## Data Availability

No new data were generated or analyzed in this study.
